# The Foreign Journals: Midwifery

**Published:** 1838-10

**Authors:** 


### MIDWIFERY.

### *On Puerperal Fever.* By Dr. A. Gruber.

Dr. Gruber recognizes different forms of puerperal fever, dependent upon different affections of different parts; he is alive to the difficulty of distinguishing these different forms from each other during life, but contends that there are two general causes of the disease, the one inflammation, the other mortification from defect of action. In inflammation of the uterus, its muscular texture becomes swollen and hard and its increased size is attributable to no want of contraction but to increased thickness of its parietes. In mortification of the uterus, on the other hand, its texture becomes thin and weak, its substance wastes away, it loses its power of contraction and its cavity is increased in size. The inflamed uterus retains a spherical form but when affected by gangrene it becomes flat or even wrinkled. Inflammation of the uterus is accompanied by pain and restlessness, but when gangrene is present there is no pain, either at the onset, or in the progress of the disease. In inflammation, too, there is great tenderness and increase of heat in the inflamed parts; in gangrene tenderness is absent and the heat is often diminished. Examination after death of those who have died of inflammation of the uterus does not always detect disease of the mucous membrane of the intestinal canal; after death from mortification, however, the mucous membrane of the stomach, of the lower part of the ileum, and of the colon on both sides is found changed, and is usually softened, loose, of a mottled white and grey colour. Lastly, in inflammation of the uterus antiphlogistic measures are of great service, in gangrene they are evidently injurious. Dr. Gruber contends that gangrene of the uterus is not necessarily the result of inflammation but often exists independently of it, as is proved both by the symptoms during life, and by the appearances after death. When the peritoneum is the seat of the disease and there is low action, the severe pain, restlessness, and anxiety, which are the chief diagnostic symptoms of the acute inflammation of the membrane, are entirely absent. The progress of the disease is always slower in this case and death does not commonly take place till the fourth or fifth week. In every case of this kind which the author observed, putrescence of the uterus was present, and the mucous membrane of the stomach and intestines was reduced to a pulp. The diseased peritoneum was of a black colour, loose, with a rough granular surface, easily detached from the subserous cellular membrane which was found in the same state. The cavity of the peritoneum contained some ounces of a dark brown fluid without any trace of cheesy or membranous deposits. This state of the peritoneum was always accompanied by gangrene of the uterus and was invariably most strongly marked in the immediate neighbourhood of that organ. With regard to the mixed character of the contents of the peritoneal cavity, Dr. G. thinks that the cheesy matter may be regarded as the product of the inflamed membrane, and the serous effusion of the uninflamed portions; the former is found wherever there have been symptoms of peritoneal inflammation during life, the latter where they have been entirely absent. We will add one other remark of Dr. Gruber that chlorosis strongly disposes to gangrene of the uterus; twelve years' experience have convinced him of the truth of this observation. The author draws no practical inferences from his views of the pathology of puerperal fever.

*Zeitschrift für die gesammte Medicin.* Band v. Heft 2.

---

### *Case of Spontaneous Rupture of the Uterus.* By M. Gendrin.

A woman, aged twenty-four, was admitted at La Pitié under the care of M. Gendrin. She was in the sixth month of pregnancy, having had a favorable accouchement of her first child three years previously. At the fifth month of her second pregnancy a slight hemorrhage came on, followed by uterine contractions. These symptoms recurred frequently and reduced her to a state of great weakness. She came into the hospital about the end of December, 1836, very pale and thin. On examination the fundus of the uterus was found to have reached the umbilicus, the back and feet were easily recognized through the abdominal parietes; the pulsations of the fœtal heart were heard on the right side; the movements of the child were felt by the hand placed on the abdomen. The head was presumed to be in the second position. The neck of the uterus was not obliterated; it was soft. Pains were felt at long intervals, and a slight muco-sanguineous discharge existed. She went on thus for several days. On the night of 2d January, 1837, violent pains came on, which were very acutely felt, and did not entirely cease between the contractions: in the morning she was sure she could not go on all day without relief; the movements of the child were no longer felt; the os uteri being scarcely at all dilated: a dose of ergot was given, an emollient enema having been previously administered; but she sunk into a state of exhaustion, and died at three o'clock in the afternoon.

*Dissection*, forty hours after death. On opening the abdomen, the body of the child was found in contact with the intestines, the back directed forwards, the feet upwards; it had escaped through a longitudinal laceration in the right side of the uterus: close to the cervix, where the head had rested, was another laceration with uneven edges, not extending through the peritoneal coat. The placenta was attached over the neck of the uterus, and was partially separated. The head and placenta alone remained in the uterus. A great quantity of fluid blood mixed with liquor amnii, and some coagula were contained in the peritoneal cavity. The structure of the womb appeared to be rather softened. The pelvis was well formed and sufficiently capacious. The fœtus appeared to be about six months and a half old; it showed no sign of putrefaction. In this case the lacerations corresponded to those parts of the uterus where the head and feet had rested.

The accident was probably caused by the long continuance of slight uterine contraction, which gradually thinned the walls of the organ until they gave way, not all at once, but by degrees. In what other way can it be explained, there being no obstacle in the conformation of the pelvis?

*Gazette Médicale de Paris. February,* 1837.

